# c-Met Modulates RPE Migratory Response to Laser-Induced Retinal Injury

**DOI:** 10.1371/journal.pone.0040771

**Published:** 2012-07-13

**Authors:** Masataka Kasaoka, Jie Ma, Kameran Lashkari

**Affiliations:** 1 Schepens Eye Research Institute, Massachusetts Eye & Ear Infirmary, Department of Ophthalmology, Harvard Medical School, Boston, Massachusetts, United States of America; 2 Department of Ophthalmology, Kurume University School of Medicine, Kurume, Japan; Rutgers University, United States of America

## Abstract

Retinal laser injuries are often associated with aberrant migration of the retinal pigment epithelium (RPE), which can cause expansion of the scar beyond the confines of the original laser burn. In this study, we devised a novel method of laser-induced injury to the RPE layer in mouse models and began to dissect the mechanisms associated with pathogenesis and progression of laser-induced RPE injury. We have hypothesized that the proto-oncogene receptor, c-Met, is intimately involved with migration of RPE cells, and may be an early responder to injury. Using transgenic mouse models, we show that constitutive activation of c-Met induces more robust RPE migration into the outer retina of laser-injured eyes, while abrogation of the receptor using a cre-lox method reduces these responses. We also demonstrate that retinal laser injury increases expression of both HGF and c-Met, and activation of c-Met after injury is correlated with RPE cell migration. RPE migration may be responsible for clinically significant anatomic changes observed after laser injury. Abrogation of c-Met activity may be a therapeutic target to minimize retinal damage from aberrant RPE cell migration.

## Introduction

Lasers have been broadly applied in our world and laser instruments are being increasingly employed in a vast variety of fields, including military, health, educational, and commercial laboratories [1]. The use of laser has been increased many folds in the military, owing to its use in laser range finders, target designator and long distance communications [2]. Even in the field of ophthalmology the use of laser has increased many folds. Along with this increase in the use of laser devices, there is also a proportionate increase in ocular exposure to laser radiation [3]–[5]. Recently, a review of military and civilian data sources in 1997 estimated that 220 confirmed laser eye injuries have occurred between 1964 and 1996 [2]. Laser injuries often cause devastating disability and significant costs to the military in terms of medical care and lost work time.

Exposure to laser can cause severe clinical ocular injuries that mostly damage the RPE layer by photothermal and photodisruptive mechanisms [3]. These laser-induced injuries can vary from scars as small as a few mm in size to full thickness macular formation (disruption of the foveal anatomy. The clinical course of retinal laser injuries is characterized by initial blurred and distorted vision, possibly followed by severe late complications, which include fibrovascular scar formation, choriodal neovascularization [4] and central vision loss [5]; [6]. Apart from injury to retinal neurons from direct exposure to laser, there are also late onset complications that arise from the excessive wound healing after the initial insult. This can lead to overt fibrosis and granulation tissue formation beyond the original confines of the injured area (known as creep). Frequently secondary migration of the scar towards the foveal center can affect final visual recovery. This has been a therapeutic dilemma in management of soldiers who have received accidental YAG laser injury [5]; [7]. Limiting the size of the scar by controlling wound enlargement and inhibiting aberrant RPE cell migration are critical factors in designing therapies for laser injury.

Hepatocyte growth factor (HGF), also known as scatter factor, originally discovered and cloned as a potent mitogen for mature hepatocytes [7], is predominantly expressed by cells of stromal origin [8], including fibroblasts, vascular smooth muscle cells and glial cells [9]–[11]. Previous studies have indicated that HGF exhibits pleiotropic biological functions in its target cells as mitogen, motagen and morphogen, and also exhibits proangiogenic and anti-apoptotic properties [12]–[15]. HGF is synthesized by mesenchyme-derived cells (namely fibroblasts), which primarily target epithelial cells in a paracrine manner through its receptor, c-Met. As the only known specific receptor for HGF, c-Met, a receptor tyrosine kinase, mediates all HGF-induced biological activities [16]–[19]. c-Met is a 190 kDa product of the met proto-oncogene composed of a 45 kDa α-chain that is disulfide-linked to a 145 kDa β-chain [20]; [21]. Stimulation of c-Met by HGF leads to receptor dimerization, which induces phosphorylation at 1349 and 1356 salient tyrosine sites and its kinase domain.

In the retina, c-Met is mainly expressed in RPE cells [22]. In response to pathologic conditions, RPE cells initiate a post-injury process and become transformed from a stationary epithelial state to a spindle-shaped, migratory and proliferative mesenchymal state, leading to the transretinal membrane formation associated with the development of proliferative vitreoretinopathy (PVR) [23]. Excessive RPE layer injury response can further deteriorate visual outcome after laser-induced injury, leading to scar formation beyond the confines of the site of injury itself and usually towards the central macula.

In this study, we devised a novel method of laser-induced injury to the RPE layer in mouse models and began to dissect the role of c-Met in the pathogenesis and progression of late stage complication of laser-induced RPE injury. We have hypothesized that c-Met is intimately involved in the migration of RPE cells as an early response to injury. We demonstrate that retinal laser injury increases expression of both HGF and c-Met, and induces phosphorylation of the c-Met suggesting activity. Using transgenic mouse models, we show that constitutive activation of c-Met induces more robust RPE migration while abrogation of the receptor reduces these responses, suggesting that modulation of the c-Met activity may influence post-injury responses to laser burns. This study identifies c-Met as a potential therapeutic target to control aberrant RPE migration and wound enlargement after laser-induced injury.

**Table 1:** Summary of mice used in this study (verified by genotyping).

Mice	Background	c-Met genome	c-Met activity
B6	C57BL/6	Homozygous c-Met receptor	Wild-type
TPR-Met	C57BL/6 × FVB/N-Tg/mtTPRmet	Heterozygous TPRmet	Constitutively active
c-Met^fl/fl^	129-C57BL/6-met^fl/fl^ × C57BL/6	Homozygous floxed c-Met	Wild-type; abrogated by Cre

**Figure 1:**
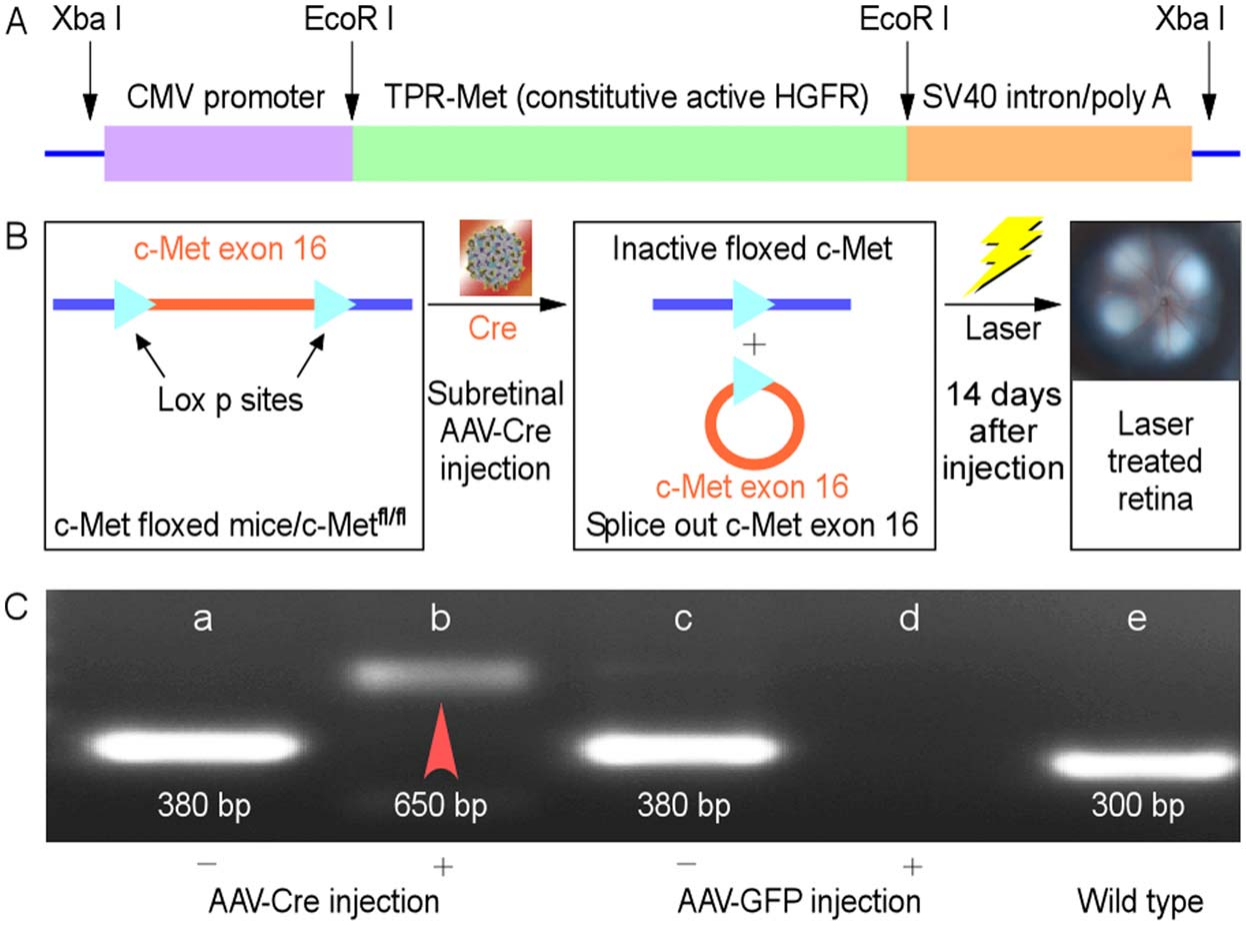
Structural diagram of *cMet* in TPR-Met mice (A) and schematic of Cre-mediated knock-out of c-Met by AAV-Cre delivered subretinally in the homozygous c-Met^fl/fl^ mice (B). (C) Before AAV-Cre injection, retina lysates of c-Met^fl/fl^ mice were prepared for genotyping by PCR reaction. A 380 bp amplification fragment was specific to the floxed allele (a and c); a 300 bp fragment to the wild-type allele (e); in AAV-Cre injected mice, a 650 bp fragment was detected specific to the deleted allele (b, indicated by a red arrowhead) while mice subretinally injected with AAV-GFP did not show the corresponding band (d).

## Materials and Methods

### Ethics Statement and Animal Models

This study was approved by the IACUC of the Schepens Eye Research Institute and in accordance to the Declaration of Helsinki for the use of animals. All experiments were performed in accordance with the association for Research in Vision and Ophthalmology Statement for the Use of Animals in Ophthalmic and Vision Research. In this study we compared three different types of mice which are detailed in Table 1. B57BL/6 (B6) mice were purchased from Charles River Laboratories (Cambridge, MA) used as a model for wild-type c-Met expression. FVB/N-Tg/mtTPRmet mice were obtained from Jackson Laboratories (Bar Harbor, ME) and backcrossed to B6 mice ×6 to produce a stable colony (C57BL/6/FVB/N-Tg/mtTPRmet) in the B6 background (TPR-Met mice). In TPR-Met mice, the extracellular domain of c-Met gene was replaced with TPR gene. This provided two strong demerization motifs and subsequent constitutive activation of the receptor in an HGF-independent manner [24]. To ensure proper c-Met expression in TPR-Met mice, SV40 splicing and polyadenylation signals were added to the structure (Figure 1A). Heterozygous TPR-Met mice used to evaluate the augmentation of c-Met activity and identified by genotyping of their tail clippings.

**Table 2:** The primers for detecting the mRNA change after the laser treatment in the retinas of C57BL/6 mice.

Genes	Primers	Sequences
HGF	Forward	5′-TTCCCAGCTGGTCTATGGTC-3′
	Reverse	5′-TGGTGCTGACTGCATTTCTC-3′
c-Met	Forward	5′-ATGAAATCCACCCAACCAAA-3′
	Reverse	5′-TCTGAATTTGAGCGATGCTG-3′
GAPDH	Forward	5′-AACAGCAACTCCCACTCTTC-3′
	Reverse	5′-CCTCTCTTGCTCAGTGTCCT-3′

To study the effects of c-Met receptor abrogation, 129-C57BL/6-met^fl/fl^ mice, a conditional knockout of c-Met, was obtained from National Cancer Institute (Bethesda, MD). These mice were also backcrossed to B6 mice ×6 to produce a stable colony in the B6 background (c-Met^fl/fl^ mice). In c-Met^fl/fl^ mice, exon 16 of the c-Met genome is flanked by lox p sites. In presence of Cre recombinase (delivered by subretinal injection of adeno-associated virus harboring Cre recombinase (AAV-Cre), floxed p sites are permanently spliced out rendering c-Met inactive (Figure 1B). To evaluate the efficiency of Cre-ligation of floxed p sites in the c-Met, retinal lysates of c-Met^fl/fl^ mice were prepared and subjected to PCR reaction, which produced a 380 bp amplification fragment specific to the floxed allele (Figure 1C, a and c) or a 300 bp fragment specific to wild-type allele (Figure 1C, e). Subretinal injection of AAV-Cre in the homozygous c-Met^fl/fl^ mice produced a 650 bp fragment specific to the deleted allele (Figure 1C, b). In contrast, no such 650 bp fragment was detected after subretinal injection of AAV expressing green fluorescent protein (AAV-GFP) (Figure 1C, d). The genotype of c-Met^fl/fl^ mice is summarized in bottom panel in Figure 1, and homozygous mice were used to evaluate the abrogation of c-Met by subretinal injection AAV-Cre.

### Procedure for Retinal Laser Injury

Mice were anesthetized with a mixture of Ketamine and Xylazine previously diluted in sterile saline at a dose of 120 mg/kg and 20 mg/kg, respectively. Only right eyes of mice were used for laser treatment. The anesthesic mixture was injected intraperitoneally using a 27G needle. Mice were kept on heat pad during and after the procedure of anesthesia. Pupils were dilated with topical application of 5% phenylephrine and 0.5% tropicamide solution. A flat glass cover slip was applied to the cornea to neutralize corneal and lenticular diopteric power. Laser burns were created using a diode laser (IRIS Medical OcuLight SLx, IRIDEX Corporation, Mountain View, CA) with a wavelength of 810 nm, spot size of 350 µm, 150 mW power for 150 ms. 12 laser spots were applied in each animal, 3 per each retinal quadrant centered around the optic nerve (Fig. 1B). For sham injections, the laser was set to “standby” and the foot pedal was depressed.

**Figure 2:**
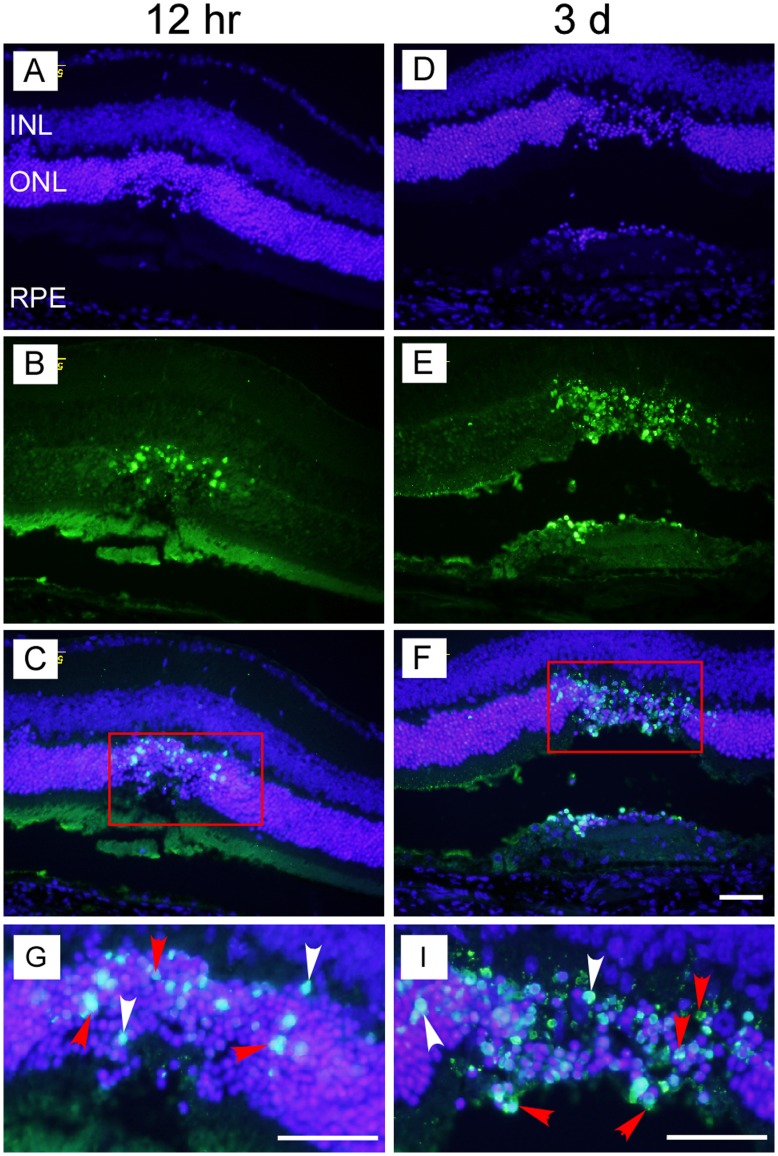
Terminal deoxynucleotidyl transferase dUTP nick end labeling (TUNEL) indicated that laser injury induced early apoptosis in the outer nuclear layer (ONL) in B6 mice. TUNEL is a common method for detecting DNA fragmentation that results from apoptotic signaling cascades. No significant morphological disorganization was observed in the retina within hours after laser burns (A, D). Nuclei in ONL exhibited signs of apoptosis about 12 hr after laser injury (B – C) and reached the apoptosis peak by day 3 (E – F, TUNEL-positive nuclei labeling with green fluorescence). The apoptotic and dead cells were indicated by red and white arrowheads, respectively (G – I). Scale bar for images: 100 µm. Abbreviations (the same in the following Figures): B6 mice, C57BL/6 mice; INL, inner nuclear layer; ONL, outer nuclear layer; RPE, retinal pigment epithelium.

**Figure 3:**
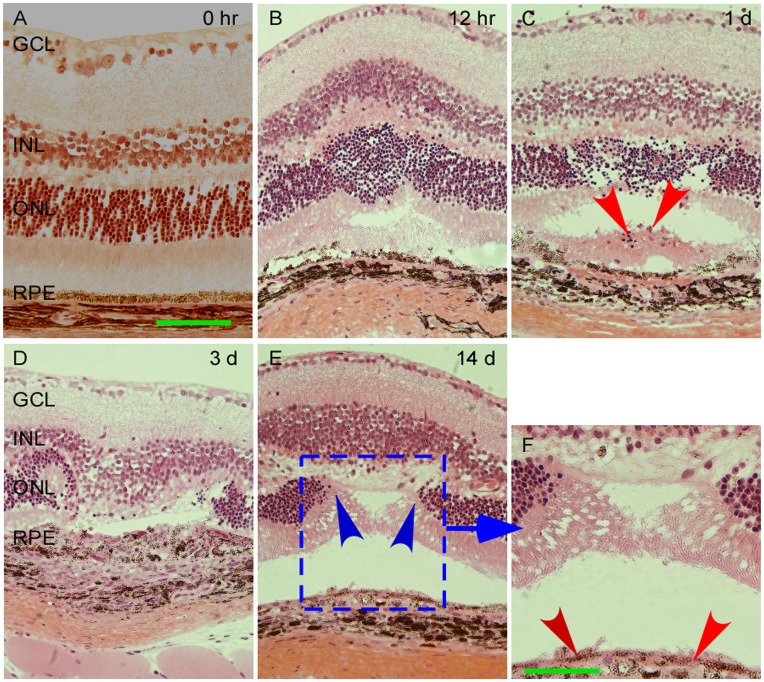
Representative images of morphological features in the retinal layer following laser burn injury in B6 mice. Tissue were embedded in paraffin and stained with hematoxylin and eosin. Eye receiving sham laser injury shows intact retina and RPE layers (A). At 12–24 hr after the laser burns, INL and ONL begin to show structural disorganization with some photoreceptors loss (B – C). RPE monolayer was disrupted and pigmented cells were observed in the subretinal space (red arrow; C). On day 3, significant photoreceptors loss was observed in conjunction within the laser-injured area (D). No photoreceptor was found in the injury area at day 14 (E, indicated by blue arrows). The RPE monolayer reformed at the wounded area suggesting reformation of a new blood-retina barrier (F, indicated by red arrows). The scale bar for all images: 100 µm. Abbreviation: GCL, ganglion cell layer.

All mice were scarified by carbon dioxide inhalation at the different intervals from 0.5 hr to 14 days after the laser treatment. Eyes were quickly enucleated and fixed in 4% paraformaldehyde for histological examination. Some fresh retinas were collected to extract the mRNA for c-Met and HGF expression.

**Figure 4:**
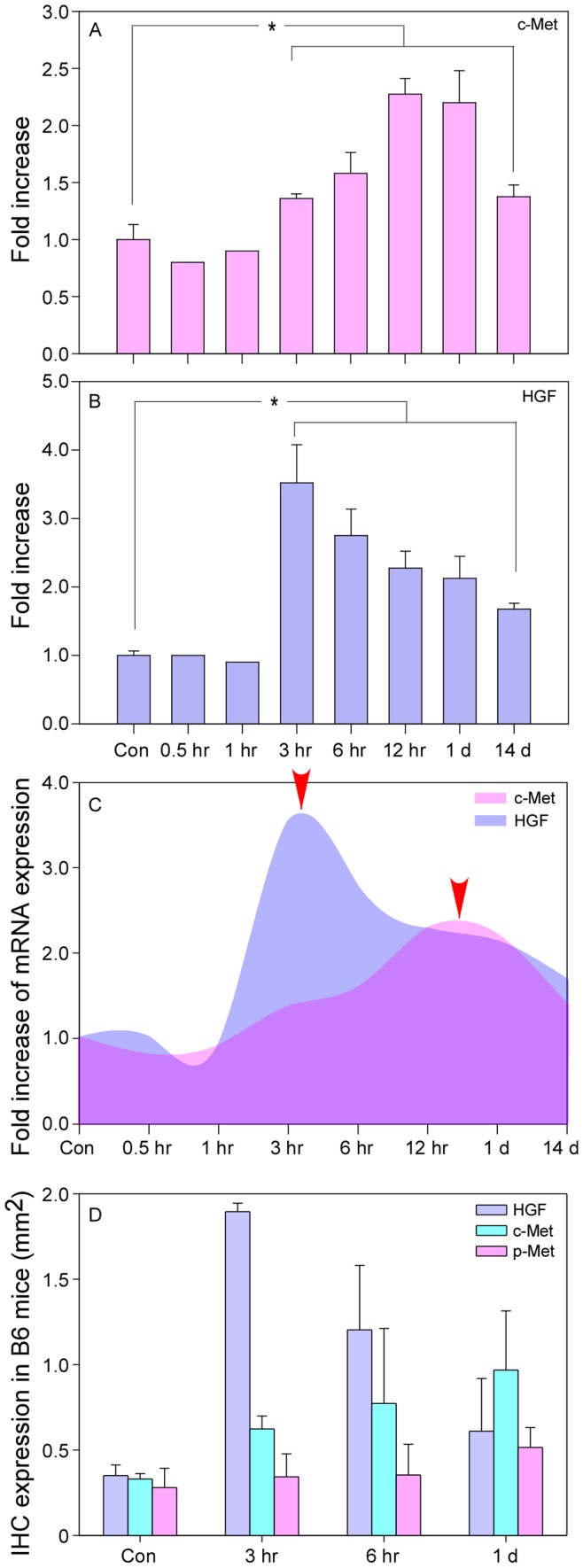
Quantified gene expression in laser-injured retinas of B6 mice. Data are presented as fold increase over sham-treated eyes and normalized to expression of GAPDH. mRNA expression of c-Met, the cognate receptor for HGF, reached its peak value around 12 hr after the laser injury (A), while mRNA level of HGF peaked at 3 hr (B) (indicated by arrows in C, respectively). A hysteresis relationship was identified between the expression of c-Met and HGF (arrowheads, C). (D) Expression of phosphorylated c-Met (p-Met) did not show any significant change over time after laser application. Abbreviations: HGF, hepatocyte growth factor; GAPDH, glyceraldehyde 3-phosphate dehydrogenase; Con, control retinas received sham laser burns; IHC, immunohistochemical staining. *P<0.05 (Mann–Whitney U test, n = 5).

**Figure 5:**
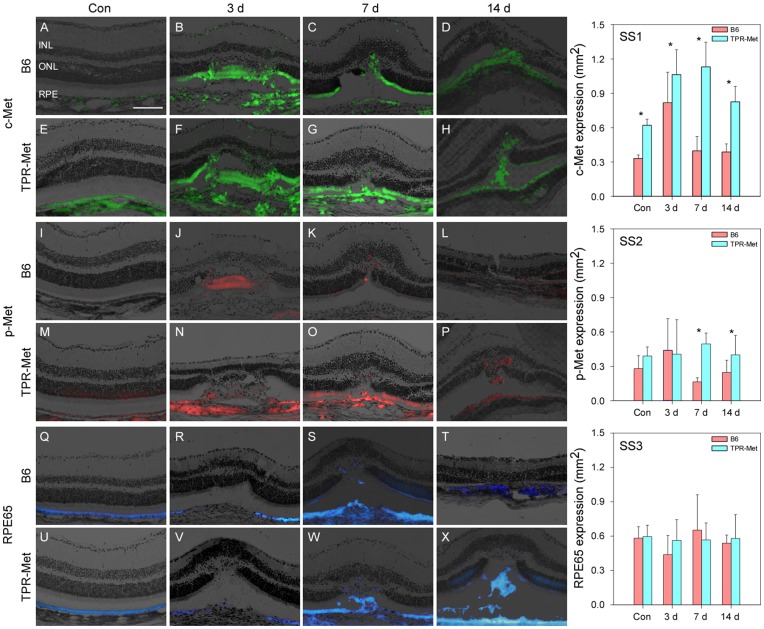
Dynamic changes in expression of c-Met, p-Met and RPE65 in the retina of B6 and TPR-Met mice after laser injury. After sham laser injury, very trace c-Met expression was detected in the control retina (A). c-Met expression was increased up to day 3 (B). On day 7 and 14, c-Met expression (C – D) decreased but remained higher than control (A). Similarly, in TPR-Met mice, c-Met expression significantly increased after laser injury (F) although obvious expression was found in sham-treated eyes (control; E). c-Met expression decreased from day 7 to day 14. On day 7, migrated c-Met positive cells were observed in the outer retina (C – D, G – H). On day 14, the disrupted RPE layer was reformed as a monolayer (D, H). Expression of (phosphorylated) p-Met was similar between B6 and TPR-Met mice (I – P). p-Met was not detected in control retina of B6 mice (I), but p-Met was detected as early as 3–6 hr after the laser injury. Expression of p-Met in the laser-treated areas obviously diminished (J) on day 3 and was almost undetected from day 7 to day 14 (K – L). Several p-met positive migrating cells were observed after day 7 (K – L). p-Met expression was found in the control retina of TPR-Met mice (M) and dramatically increased on day 1 after the laser injury (N – P). Some migrated cells were detected from day 7 to day 14 (O – P). Expression of p-Met in TPR-Met mice after laser treatment was higher than in B6 mice (I – P). Expression of RPE65 in B6 mice on day 7 after laser treatment was significantly higher than other conditions (S) but there was no difference between control, day 3 and day 14 (Q – R, T). In TPR-Met mice, RPE65 expression slightly decreased after laser injury on day 1 compared to control (U – V), but significantly increased from day 7 to day 14 (W – X). The RPE layer in B6 and TPR-Met mice showed disorganized morphology after laser burns up to day 3 but started to reform on day 7 and completely reformed on day 14. The expression of c-Met in B6 and TPR-Met mice rapidly increased after the laser injury (SS1). However, p-Met did not show obvious changes after the laser burns (SS2). Expression of RPE65 in laser-treated B6 and TPR-Met mice was quite similar between B6 and TPR-Met mice at different time point (SS3). The scale bar for all images: 100 µm. * P<0.05, independent samples *t*-test.

### TUNEL, Pigment Bleaching and Immunohistochemistry

After fixing overnight in 4% paraformaldehyde, eyeballs were transferred into PBS buffer with 30% sucrose for one hour and processed for either paraffin or frozen sections at a thickness of 8 µm. Terminal deoxynucleotidyl transferase dUTP nick end labeling (TUNEL), a common method for detecting DNA fragmentation that results from apoptotic signaling cascades, was used to confirm areas of laser damage. To this end, cryosections of the retina of B6 mice were stained with *in situ* Cell Death Detection Kit (Roche, Mannheim Germany).

Paraffin sections for IHC staining were warmed overnight, depraffinized with xylene, taken through serial alcohol dilutions and hydrated to distilled water. Sections were bleached for melanin pigment using an established protocol [25]. Briefly, sections were oxidized by incubation in 0.25% aqueous potassium permanganate for 30 min, washed in distilled water and bleached in 5% oxalic acid until white. Sections were then washed in PBS and subjected to immunohistochemistry using the Vectastain ABC kit with alkaline phosphatase method and resolved with Vector Red (Vector laboratories, Burlingame, CA). Primary antibodies included HGF (H-145, Santa Cruz Biotechnology, Santa Cruz, CA), c-Met (SP260, Santa Cruz Biotechnology), phospho-c-Met (07–810, Upstate Biotechnology, Temecula, CA) and RPE65 (MAB5428, Chemicon, Temecula, CA). Sections were examined under an IX51 Olympus inverted fluorescent microscope (Olympus Corporation, Tokyo, Japan) both under visible light and epifluorescence for better detection of the highly fluorescent rhodamine Vector Red pigment. For better visualization, a grey-scale fundus image was sandwiched with its corresponding fluorescence image, which was itself assigned an arbitrary color (green, blue or red).

**Figure 6 pone-0040771-g006:**
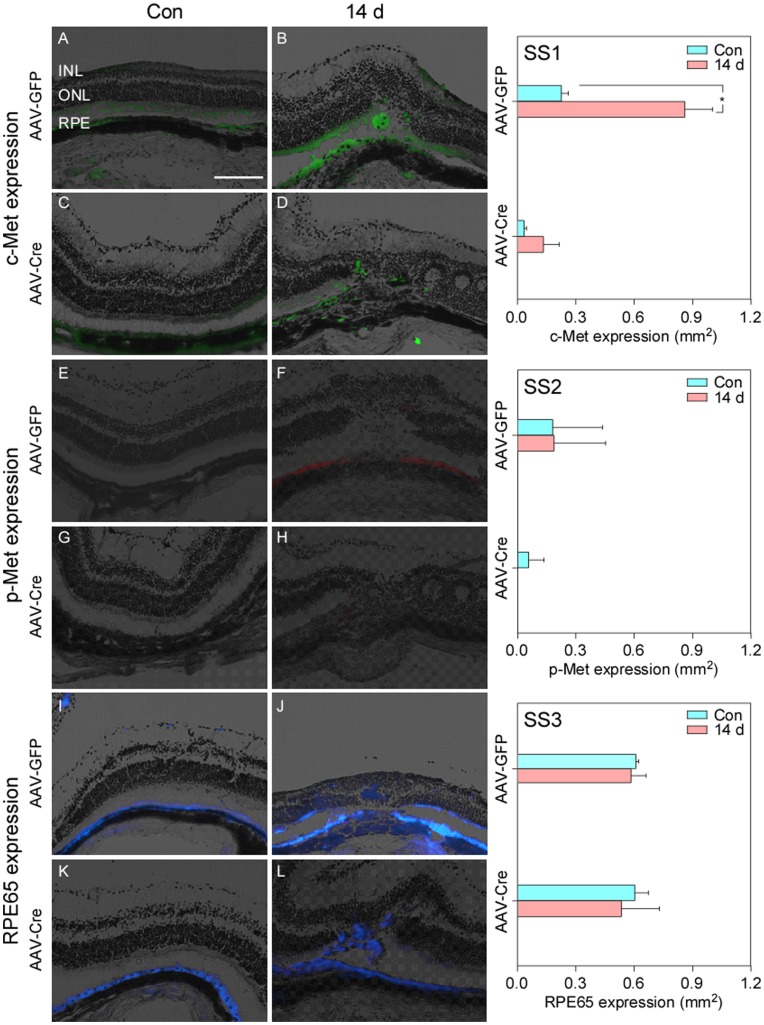
c-Met, p-Met and RPE65 expression in c-Met^fl/fl^ mice 14 days after AAV-GFP and AAV-Cre injections, respectively (A, C, E, G, I and K) without laser burns; and after laser burns (B, D, F, H, J and L). Mice were scarified on day 14 after laser application (total day, 28). In AAV-GFP injected mice, laser burns induced an expected increase in c-Met and p-Met (B vs. A; E vs. F). There was no detectable c-Met or p-Met expression was seen after subretinal AAV-Cre injection (C and G). Less c-Met and no p-Met expressed were observed even after laser injury (D and H). RPE65 expression was not affected by subretinal injection of AAV-GFP or AAV-Cre injection (I – L). There were more migrated RPE cells in ONL in AAV-Cre injected (L) compared to AAV-GFP injected mice (J). There was very limited c-Met and p-Met detected in AAV-Cre injected eyes before and after laser injury (SS1– SS2); in AAV-GFP injected eyes, c-Met expression expectedly increased after laser treatment (SS1) (* P<0.05, independent samples *t*-test). RPE65 expression was not affected by either AAV-GFP or AAV-Cre injection (SS3).

**Figure 7:**
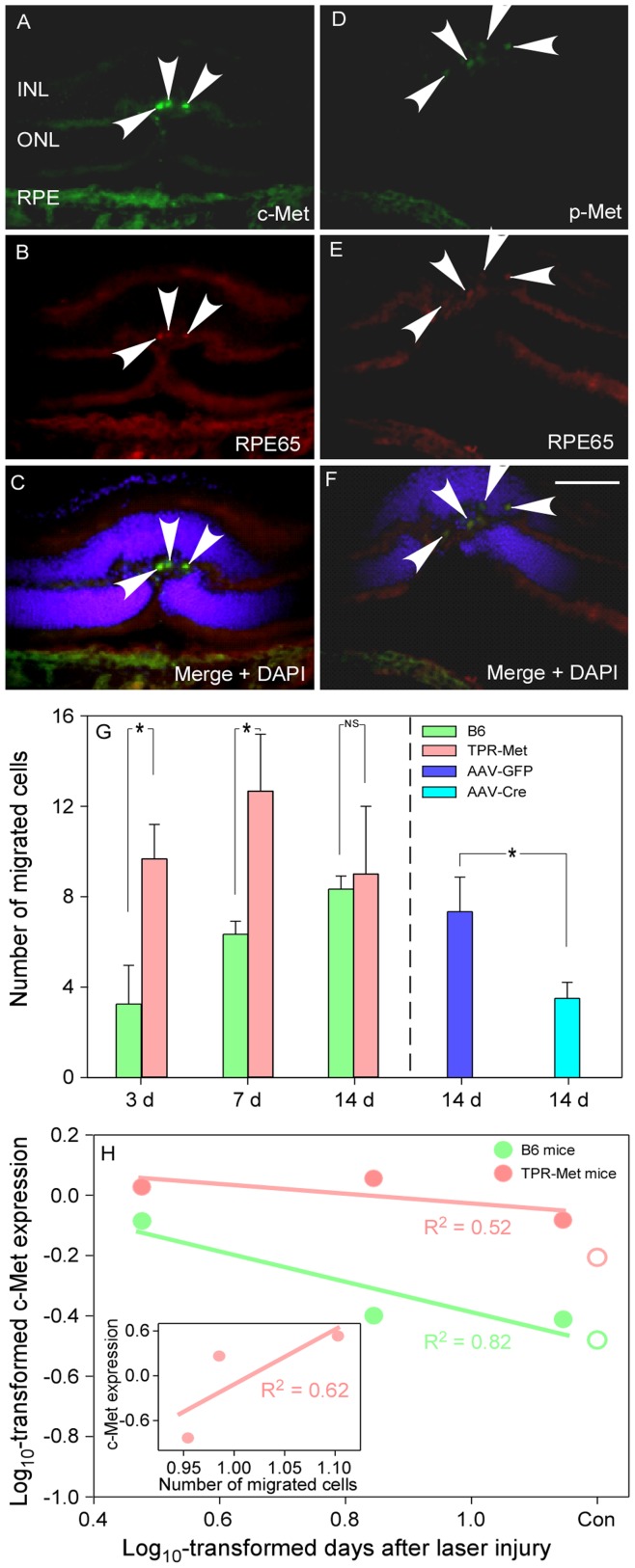
Migration of RPE cells into outer retina 7 days after to injury in B6 mice. RPE cells were observed to migrate into ONL and expressed both c-Met and p-Met (A – E); RPE65 expression confirmed that migrating cells were indeed RPE (C and F). RPE migration was observed as early as 3 days after injury. More robust RPE migration was observed in TPR-Met mice (left side in G panel). In AAV-Cre injected mice, significantly fewer migrating cells were found compared with their AAV-GFP injected counterparts (right side in G panel, * P<0.05, independent samples *t*-test). These observations indicate that higher c-Met expression could induce more RPE cells to migrate. Significant linear association was confirmed between c-Met expression and duration of laser injury, specifically from day 3 to day 14 in both B6 mice (y = −0.50× +0.11, R^2^ = 0.82) and TPR-Met mice (y = −0.16× +0.13, R^2^ = 0.52) (H). On day 14 after the laser injury, expression of c-Met in both mice gradually decreased back to baseline (control level, open circles on the right side). But the process in B6 mice may be faster (slope value, -0.50) than in TPR-Met mice (slope value, -0.16). In addition, a significant linear regression was found between the concentration of c-Met and the number of migrated RPE cells in the c-Met over-expressed TPR-Met mice (y = 0.83× +1.02, R^2^ = 0.62) (inserted graph in H panel, the values on x-axis and y-axis were log_10_-transformed according to the original measurements). These findings strongly suggest that higher levels of c-Met expression could induce more RPE cells to migrate. The scale bar for all images: 100 µm.

**Figure 8:**
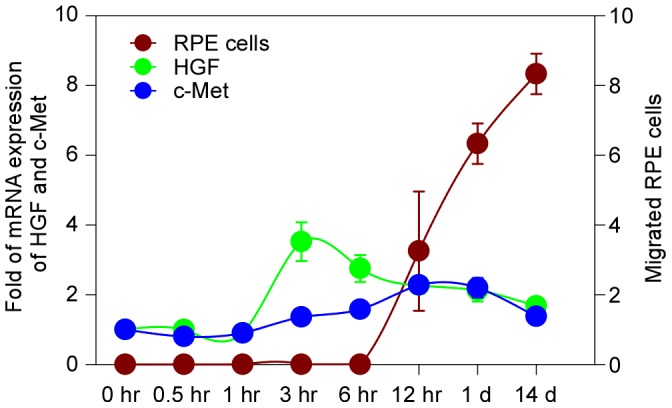
The expression of HGF and c-Met and the migration of RPE cells in B6 mouse after the laser-induced injury. We presume the accumulation of HGF expression might be able to trigger the expression of c-Met. Meanwhile, the c-Met expression positively affected the RPE cell migration after the laser injury.

### Effect of Laser Injury on Expression of c-Met and HGF in B6 Mice

To determine whether laser injury affected expression of c-Met and HGF in the RPE monolayer and outer retina, laser-induced retinas of B6 mice were collected at the following time points, 0, 0.5, 1, 3, 6, 12 hr, 1 day and 14 days (5 individuals per group). Total mRNA was extracted using RNA 4 Aqueous kit (Ambion Inc., Austin, TX). Reverse transcriptase reaction was performed for each mRNA sample using Retroscript kit (Ambion Inc., Austin, TX). 1 µg of total mRNA was used as template to synthesize first-strand complementary DNA (cDNA). RT-qPCR was performed with 3 independent repetitions using the API Prism 7900HT Sequence Detection system (Applied Biosystems, Foster city, CA) according to the instruction of SYBER Green PCR Master Mix (Applied Biosystems, Foster City, CA). The reaction program included 2 min at 50°C, 10 min at 95°C, 40 cycles for 15 s at 95°C and 60 s at 60°C. The parameter threshold cycle was designed as the fractional cycle number, at which the fluorescence signals were generated during each PCR cycle. c-Met and HGF expression was calculated from the standard curve; quantitative normalization in each sample was performed using the expression of the glyceraldehyde-3-phosphate dehydrogenase (GAPDH) as an internal control using the delta-delta method [26]. Data were presented as fold change over control. The sequences of the primers are summarized in Table 2.

### Subretinal Injection of Adeno-associated Virus (AAV) in Homozygous c-Met^fl/fl^ Mice

AAV-Cre and AAV-GFP (serotype 2) were constructed and supplied by the Harvard Gene Intiative Core (Boston, MA). AAV vectors were previously purified and titrated to about 1×10^10^ Tu/ml for both AAV-Cre and AAV-GFP. Homozygous c-Met^fl/fl^ mice were injected either with a mixture of AAV-Cre/AAV-GFP (ratio 9∶1) or AAV-GFP using a trans-scleral approach into the subretinal space under direct observation. The small amount of AAV-GFP in the AAV-Cre/AAV-GFP mixture induced a low-level background green fluorescence that can be detective in vivo by epifluorescence microscopy.

To perform this injection, a silk suture was penetrated through the upper eyelid. The eyelid was gently retracted and the eyeball was projected out from the eye socket to improve exposure. A small O-ring was placed on the eye. The pupil was dilated with one drop of each 2.5% phenylephrine and 0.5% tropicamide. Gonak (Akorn, Inc., Buffalo Grove, IL) was applied over the O-ring to make an optical connection for visualization of the fundus. A sclerotomy (puncture hole) was made in the posterior portion on the wall of the eye using a 31G needle. A micro-glass pipette was used to deliver 2 µl of AAV solution through the sclerotomy into the subretinal space. Mice receiving subretinal injection of AAV-GFP served as controls for mice receiving AAV-Cre.

Analgesic buprenex (2 mg/kg) was administererd subcutaneously to mice before procedure and every 12 hr for 2 days. Antibiotic ophthalmic ointment (Vetropolycin, Pharmaderm, Melville, NY) was applied to the eyes three times daily for 2–3 days postoperatively. Two weeks after subretinal injection, c-Met^fl/fl^ mice were subjected to laser treatment. The injected mice (5 individuals in each group) were scarified on day 14 after laser treatment and examined expression of c-Met, p-Met and RPE65 in the outer retina and RPE layer.

### Quantification on the IHC Staining, Cell Migration and Data Analysis

The respective expression areas (mm^2^) of c-Met, p-Met, HGF and RPE65 in the IHC stained slices were measured with ImageJ 1.46d (resolution at 300 pixels/mm). These included comparison of quantified measurements. Migrated cells in ONL and up to INL on the IHC stained sections were also manually counted using the image processing and analysis program ImageJ (National Institutes of Health, Bethesda, MD). One-Way ANAVO, Mann–Whitney U test and independent samples *t*-test were used to compare differences in the expression of aforementioned markers and the number of migrated cells. To establish linear regressions, log_10_-transformation was performed to normalize data and applied to the expression of c-Met, the days after the laser treatment and the number of migrated cells in B6 and TPR-Met mice. The data were presented as mean ± SD. All specific analyses and regressions were performed in SigmaPlot 11.0. The level of significance was set at 0.05.

## Results

### Cell Apoptosis and Histological Changes Induced by Laser Burns

After laser injury, the retinal wound area appeared as creamy white spots (Figure 1B, right side). Although no obvious morphological disorganization of retina was found at early stage after the laser burns (Figure 2A and 2D), apoptotic cells were detected in seemingly injured retinas. Apoptotic cells in ONL were detected by TUNEL as early as 12 hr after laser injury (Figure 2B – C). On day 3, some dead cells were found in RPE layer and more dead cells were detected in ONL (Figure 2E – F). The typical apoptotic and dead cells were indicated by red and white arrowheads, respectively (Figure 2G – I). RPE layer appeared intact before the laser burns (Figure 3A). Laser burns disrupted the RPE monolayer resulting in aberrant migration of RPE cells to the ONL at the site of laser-induced injury. At 12–24 hr after laser burns, the ONL began to exhibit structural disorganization with some photoreceptors loss as well (Figure 3B – C). On day 3, most of the photoreceptors had disappeared in the laser injured areas (Figure 3D). No photoreceptors were observed in the injured areas on day 14 (indicated by blue arrows in Figure 3E) where a scar had formed (Figure 3F). However, RPE cells had settled down and aligned to form a new monolayer at the wounded area, suggesting that a new blood-retina barrier had reformed (indicated by red arrows in Figure 3F).

### Quantified c-Met and HGF Expression in B6 Mice

The changes in the respective mRNA levels of c-Met and HGF in response to retinal laser burns were quantified in B6 mice using RT-qPCR. Mice received either sham or laser photocoagulation and were sacrificed at serial time points, ranging between 0.5 hr and 14 days. RT-qPCR results were presented as ratios of laser-treated and sham-treated retinas after being normalized to the expression of GAPDH.

There were no detectable changes in respective expressions of c-Met and HGF mRNA in B6 mice in the first 3 hr after the laser injury (Figure 4A – B). The mRNA level of c-Met reached its peak expression at 12–24 hr; on day 14, the mRNA level of c-Met was statistically higher than that of the control (all *P*<0.05, Mann–Whitney U test) (Figure 4A). At 3 hr after the laser injury, the mRNA level of HGF dramatically increased, but gradually decreased thereafter, but remained significantly higher on day 14 as compared to sham-treatment (all *P*<0.05, Mann–Whitney U test) (Figure 4B).

Interestingly, the mRNA level of c-Met did not show a simultaneous increase with mRNA level of HGF. While HGF mRNA peaked at 3 hr after the laser injury, c-Met mRNA peaked at 12 hr (indicated by arrowheads in Figure 4C, respectively). The mRNA levels of HGF and c-Met were similar between 12 and 24 hr but HGF mRNA remained constantly higher than that of c-Met during the whole time period (Figure 4C). The hysteristic phenomenon on the mRNA expression of these two genes may indicate that c-Met, a receptor for HGF, would not be triggered simultaneously by the expression of HGF. The accumulation of HGF mRNA may be necessary to trigger c-Met mRNA expression. This hysteristic phenomenon on the expression of c-Met and HGF was also confirmed by IHC staining (Figure 4D) (More detailed IHC images are shown in Figure 5–6). ImageJ was applied to measure the area of marker expression (c-Met, HGF and p-Met) on stained sections. These measurements indicate that HGF protein expression rapidly increased (1.90±0.05×mm^2^) and was significantly higher than the expression of c-Met or p-Met (Independent samples *t*-test, all *P*<0.05) at 3 hr after the laser injury. c-Met protein expression responded much slower and peaked at one day after injury (0.97±0.35 mm^2^). Expression of p-Met was constant within the first 24 hr (range: 0.28–0.51 mm^2^) after laser injury (independent samples *t*-test, all *P*<0.05) (Figure 4D).

### Expression of c-Met, p-Met and RPE65 in B6 and TPR-Met Mice

Expression of c-Met, p-Met and RPE65 showed dynamic changes in the retinas of B6 and TPR-Met mice at different time points after laser injury. In B6 mice, very limited c-Met expression (0.33±0.03 mm^2^) was detected in the control retina (Figure 5A). c-Met expression was higher in TPR-Met mice (0.62±0.05 mm^2^) (Figure 5E). After laser application, c-Met expression increased up to day 3 (0.82±0.27 mm^2^) (Figure 5B). On day 7 and 14, its expression (0.46±0.91 mm^2^ and 0.30±0.04 mm^2^, Figure 5C – D) decreased to near baseline and sham-lasered levels (independent sample *t*-test, both *P*>0.05). The increase of c-Met expression (Figure 5B – D) was coincident with the RT-qPCR analysis (Figure 4A).

In TPR-Met mice, c-Met expression significantly increased 3 days after laser injury (Figure 5F) compared to the control (1.06±0.22 mm^2^ vs. 0.62±0.05 mm^2^, independent samples *t*-test, *P*<0.05, Figure 5E). c-Met expression of TPR-Met mice began to decrease from day 7 to day 14 (1.13±0.22 mm^2^ and 0.83±0.13 mm^2^, Figure 5G – H) but was still higher than control (independent samples *t*-test, both *P*<0.05, Figure 5E). Generally, the expression of c-Met in TPR-Met mice was higher than in B6 mice (independent samples *t*-test, *P*<0.05, Figure 5SS1). Prior to laser injury, there was low but detectable p-Met expression in untreated retinas of TPR-Met mice (0.39±0.08 mm^2^, Figure 5 M) and significantly less in B6 mice (0.28±0.11 mm^2^, Figure 5I). After laser injury, expression of p-Met in B6 mice was subtly increased (0.44±0.28 mm^2^, Figure 5J) on day 3 and then returned to control levels from days 7 to day 14 in B6 mice (0.17±0.04 mm^2^ and 0.25±0.11 mm^2^, Figure 5K – L). In TPR-met mice, p-Met expression on day 3 after laser injury (range: 0.41±0.40 mm^2^) was quite similar to control levels (0.39±0.08 mm^2^) (independent samples *t*-test, *P*>0.05, Figure 5M – P). Quantified measurements of p-Met expression are presented in Figure 5SS2.

The expression of RPE65 in B6 mice after laser injury did not show any significantly changes among different time points (range: 0.44–0.65 mm^2^, One-Way ANOVA, all *P*>0.05) (Figure 5Q – T and Figure 5SS3). In TPR-Met mice, RPE65 expression was very stable after laser injury (0.56–0.58 mm^2^) compared to control (0.60±0.10 mm^2^, Figure 5U – V) and no differences were found among them (One-Way ANOVA, all *P*>0.05, Figure 5U – X and Figure 5SS3).

### Responses of RPE Monolayer to Laser Injury in c-Met^fl/fl^ Mice

In this study, the mixture of 9∶1 AAV-Cre/AAV-GFP or AAV-GFP was subretinaly injected into homozygous c-Met^fl/fl^ mice. One group of c-Met^fl/fl^ (n = 5) received only AAV-GFP injection without laser burns (Figure 6A, 6E and 6I). In these AAV-GFP injected mice, both c-Met and RPE65 were detectable (0.23±0.04 mm^2^ and 0.59±0.08 mm^2^, respectively) and p-Met expression was very low (0.13±0.18 mm^2^). A second group of c-Met^fl/fl^ mice (n = 5) received AAV-Cre/AAV-GFP (9∶1) injection without laser burns. In these mice no c-Met or p-Met was detected but RPE65 was expressed normally (0.03±0.01 mm^2^, 0.04±0.06 mm^2^ and 0.61±0.07 mm^2^, respectively) (Figure 6C, 6D and 6K). A third group of c-Met^fl/fl^ mice received subretinal injection of AAV-GFP followed by laser injury (2 weeks later). 14 days after the laser injury c-Met and p-Met expression of AAV-GFP injected mice increased comparing their control counterparts (0.86±0.14 mm^2^ and 0.14±0.19 mm^2^, respectively) (Figure 6B and Figure 6F). However, RPE65 expression remained similar controls (0.61±0.01 mm^2^, 0.59±0.08 mm^2^, respectively (Figure 6I – J). A fourth group of c-Met^fl/fl^ mice received subretinal injection of AAV-Cre/AAV-GFP (9∶1) followed by laser injury (2 weeks later). In these mice the expression of c-Met was very limited (0.13±0.08 mm^2^) (Figure 6D) and no p-Met was detected (Figure 6H). RPE65 expression was not affected (0.54±0.20 mm^2^) (Figure 6L). Overall, laser injury in AAV-Cre injected c-Met^fl/fl^ mice induced a relative (statistically non-significant) increase in c-Met expression compared to controls (Figure 6SS1) without any detectable p-Met expression (Figure 6SS2) or changes in RPE65 expression (Figure 6SS3).

### Role of c-Met on RPE Cell Migration after Laser Burns

Cryosections of B6 mouse retina were used to count the number of migrated cells in response to the laser injury. On day 7 after injury, migrated cells were observed in the ONL. IHC confirmed that these cells were indeed RPE. Furthermore, it indicated that c-Met (green, Figure 7A), p-Met (green, Figure 7D) and RPE65 (red, Figure 7B and 7E) were detected in migrated cells. Co-staining also confirmed that the migrated cells originated from RPE layer (Figure 7C – F), and RPE65 was used to confirm that migrated cells were indeed RPE cells in the three types of mice.

For quantifying migrated RPE cells after laser injury, cells were manually counted on the IHC staining slices using ImageJ. In B6 mice, RPE cells were identified in the ONL on day 3 after the laser injury (3±2 cells), and more cells were identified on days 7 (6±1 cells) and 14 (8±1 cells) (green bars, Figure 7G). In TPR-Met mice with laser injury, more migrated RPE cells (red bars, Figure 7G) were observed on days 3 (10±2 cells) and 7 (13±3 cells) compared with B6 mice (independent samples *t*-test, both *P*<0.05, at left side of Figure 7G). However, the number of migrated RPE cells in TPR-Met mice began to decrease (9±3 cells) on day 14; this response was similar to B6 mice (independent samples *t*-test, *P*>0.05, Figure 7G). In contrast, p-Met expressing migrated cells were quite rare in both B6 mice (3±1 cells) and TPR-Met mice (5±2 cells) (Figure 5P and Figure 5L) after the laser treatment. In c-Met^fl/fl^ mice, few c-Met positive cells (4±1 cells) were found in ONL of AAV-Cre injected mice (Figure 6D and Figure 7G), but more migrated cells (8±2 cells) were found in AAV-GFP injected mice (independent samples *t*-test, *P* = 0.03) (Figure 6B and Figure 7G). No p-Met positive migrated cell was detected after AAV-Cre injection and laser treatment (Figure 6F and 6H).

To investigate the temporal relationship for c-Met expression after laser injury in B6 and TPR-Met mice, log_10_-transformation was applied to both the days and c-Met expression from day 3 to day 14. Linear regression analysis confirmed that c-Met expression was significantly negatively related with the time (days) after the laser treatment in B6 (y = −0.50× +0.11, R^2^ = 0.82) and TPR-Met mice (y = −0.16× +0.13, R^2^ = 0.52) (Figure 7H). Furthermore, the tread line indicated that the change of c-Met expression in B6 mice more rapidly returned to control levels than in TPR-Met mice (Figure 7H). This observation suggests that laser injury is able to induce higher and longer c-Met expression in the TPR-met mice.

Interestingly, a significant linear relationship was confirmed between the expression of c-Met and the migration of RPE cells in TPR-Met mice (y = 0.83× +1.02, R^2^ = 0.62, insert graph in Figure 7H). In another word, migration of RPE cells was positively associated with the concentration of c-Met expression. Therefore, in TPR-Met mice, laser injury induced constitutively high c-Met expression, which induced more RPE cells to migrate to the inner layers of retina. Contrarily, relative abrogation of c-Met expression in c-Met^fl/fl^ mice tapered RPE migration after laser injury.

## Discussion

### Laser Injury Model as a Clinically Relevant Model of Retinal Damage

Lasers have been broadly applied in our world and laser instruments are being increasingly employed in a vast variety of fields, such as military, health, educational, and commercial laboratories [1]. The use of lasers has been increased many folds in the military, owing to its use in laser range finders, target designator and long distance communications [2]. Even in the field of ophthalmology the use of laser has increased many folds. Along with this increase in the use of laser devices, there is also a proportionate increase in ocular exposure to laser radiation [3]–[5].

Laser-induced retinal alternations characterized to date indicate that with increased energy, damaged areas extend to outer segment layers of the retina in addition to the RPE, which itself is considered the primary site of absorption [27]. These injuries mostly damage the RPE layer by photo-thermal and photo-disruptive mechanisms [3], which coincide with our findings (Figure 3C). In our study, the RPE layer began to disorganize as early as 12 hr after the laser injury. The disorganization became quite severe on day 1 post-injury (Figure 3). After the disorganization in RPE layer, some RPE cells were observed to migrate toward the ONL, as confirmed in our study by IHC (Figure Figure 5–7). Visual loss after laser injury is related to the location of the laser damage. For example, laser injury to the fovea would likely lead to an immediate and significant vision loss. Parafoveal laser lesion may involve the fovea temporarily through inflammation and edema, resolving over days to weeks, or may spread to the fovea through secondary neuronal cell damage (creep), causing permanent defects [1]; [4].

### RPE Cell Migration and Proliferation

RPE cell migration and proliferation are believed to play a role in expansion of laser scars and pathogenesis of PVR [28]. Retinal injuries from laser exposure can have variable but potentially devastating effects ranging from mild discomfort and dazzling to scaring and complete loss of central vision. The direct effect of these injures can not only lead to loss of photoreceptors and other neuronal cell types, but also to aberrant scar formation in the retina. Our findings confirmed that the photoreceptors are largely lost from day 1 after the laser injury (Figure 3 and Figure 7). Very frequently, parafoveal scars caused by laser injury expand to include previously uninvolved areas primarily due to aberrant RPE migration. If this migration and its ensuing scar formation involve the foveal center, central visual loss will ensue. Any approach to limit RPE migration through receptor abrogation or inhibition of migratory mechanisms may potentially limit this damage.

Human vitreous contains not only mitogens for RPE cells but also factors that mediate their migration. Clinically, the appearance of RPE cells in the vitreous may be a consequence of injury or rhegmatogenous retinal detachment in which these cells now become exposed to the vitreous [28]. However, RPE cells would not proliferate in the vitreous unless there is a break in the blood-ocular barrier that would allow serum including albumin and other factors to access the vitreous [28]; [29]. Extremely low levels of coherent radiation can produce ultrastructural alterations in sensory retina without apparent change in the RPE [27]; [30]. More severe injuries such as those caused by Nd:YAG laser can induce the migration of RPE cells when the blood vessels are broken to cause serum leakage [28]; [29]. In this setting, RPE cells can be induced to transdifferentiate and migrate.

In vitro studies have shown that RPE cells can transdifferentiate to either neurons or lens cells in culture [31]. An in vivo study of retinal regeneration provided evidence that the association of RPE cells with the retinal vasculature is an important step in transdifferentiation [32]. In our present study, cells expressing RPE65 were found in ONL of B6 mice on day 7 after the laser injury (Figure 7O) suggesting its RPE origin. In TPR-Met mice with continuatively active c-Met, more RPE65 positive cells were observed on days 7 and 14 after the laser burns compared to their B6 counterparts (Figure 7S – T and Figure 7W – X). Previous studies found that vitreous from eyes treated with each of the above modalities caused significant stimulation of RPE migration while control vitreous and saline-injected vitreous caused very limited RPE stimulations [29]. Our findings indicate that constitutive activation of c-Met induced stronger RPE cell migration from the laser-induced injury site to outer layer of the retina; similarly, abrogation of c-Met activity in the c-Met^fl/fl^ floxed mice reduced RPE migration into the wounded sites.

### Role of c-Met and its responses to retinal laser injury

c-Met participates in cell growth and migration during embryonic development, and plays a significant role in skin regeneration process. The c-Met protein also known as HGF receptor encodes for a thyrosine kinase receptor which is activated by HGF. Birchmeier et al. showed that receptor-type tyrosine kinases are critical in regulating epithelial differentiation and morphogenesis [33]. Schmidt et al. in 1995 performed similar studies on the effects of HGF knockout in embryonic mice and reported that HGF plays a significant role in developing several epithelial organs [34]. Furge et al. in 2003 proved the role of c-Met in Ras-mediated tumorigenecity and metastasis and suggested that HGF-Met signal inhibitors may have important therapeutic value for the treatment of metastatic cancers [35]. In addition, some previous studies have confirmed that c-Met is overexpressed in a variety of tumors in which it plays a central role in malignant transformation [36].

HGF is the only known ligand for the c-Met receptor. c-Met is normally expressed by cells of epithelial origin [34] while expression of HGF is restricted to cells of mesenchymal origin. Upon HGF stimulation, c-Met induces several biological responses that collectively give rise to a program known as invasive growth. We presume that accumulation of the HGF could initiate stimulation to the expression of c-Met. Our analyses indicate that there was a hysteresis between the peak expressions of HGF and c-Met (Figure 4C and Figure 8). This may indicate that as the receptor of HGF, the activation and expression of c-Met could only be triggered by a certain concentration of HGF. In our present study, expression of c-Met in constitutively activated TPR-Met mice was higher than in B6 mice (on days 3 and 7; Figure 5SS1). Although the exact mechanism between c-Met expression and RPE cell migration is still unclear yet, according to our findings, augmentation of c-Met expression could positively affect the migration of the RPE cells after the laser injury (insert graph in Figure 7H). In addition, in control mice (B6) we also made a similar observation that the accumulation of c-Met expression probably leads to the increase in RPE cell migration after the laser injury (Figure 8).

There are situations in vivo in which RPE cells may migrate- development [34]; [37] and wound healing [38] (including PVR and in diseases such as age-related macular degeneration [39]–[41]). After retinal laser injury, photon absorption primarily by the melanin pigment causes thermal damage to the retina. This absorbed laser light is densely concentrated in RPE cell layer and focally absorbed in the choroid [6]. This process may lead to leakage of serum, which can significantly release HGF, activate c-Met receptors and induce migration of RPE cells [28]; [29]. Activation of c-Met after laser injury may induce RPE cells to migrate and transdifferentiate. This study describes two key observations that relate the role of c-Met on RPE cells to laser injury responses. (1) Constitutive activation of c-Met via the TPR-met receptor increased both the expression of c-Met protein on RPE cells and caused more robust RPE migration into outer retina. (2) Abrogation of the c-Met receptor using the Cre-lox system reduced RPE migration without affecting RPE65 expression. If limiting RPE migration is a critical factor to limit wound growth and creep, then it may be possible to limit aberrant wound responses through inhibition of c-Met activity.

### Conclusions

Clinically, RPE cells can migrate anywhere in the retina. RPE cell migration may be mediated through the activation of the c-Met receptor. In this scenario, c-Met activation induces transdifferentiation of RPE cells and its migration across the all retinal surfaces. In response to retinal laser injury, c-Met receptor system is activated through release of HGF and is intimately involved in the responses of RPE to laser injury. This is supported by the observation that constitutive activation of c-Met increased RPE migration into the retina and abrogation of the receptor diminished RPE cell migration. Therefore, control of c-Met activity may be a future therapeutic target to minimize retinal damage that may ensue after laser injury.
